# Resolution of Mental Nerve Paresthesia Associated With a Large Periapical Lesion Following Nonsurgical Endodontic Retreatment: A Case Report With 6‐Year Follow‐Up

**DOI:** 10.1155/crid/8800002

**Published:** 2026-10-06

**Authors:** Ammar AbuMostafa

**Affiliations:** ^1^ Department of Restorative Dentistry College of Medicine and Dentistry Riyadh Elm University Riyadh Riyadh Province Saudi Arabia riyadh.edu.sa

**Keywords:** healing, large lesion, paresthesia, retreatment

## Abstract

Mental nerve paresthesia of odontogenic origin is an uncommon yet clinically significant presentation. Although malignancy remains the primary concern in the differential diagnosis of lower lip numbness, large periapical lesions of endodontic origin represent an underrecognized but treatable etiology. This case report describes the successful resolution of mental nerve paresthesia associated with a large periapical lesion through nonsurgical endodontic retreatment, with follow‐up extending over 6 years. A 30‐year‐old systemically healthy male presented with a 1‐week history of numbness affecting the left lower lip and chin. Clinical examination of tooth 36 revealed a defective composite restoration overlying a previously treated root canal. Neurosensory assessment demonstrated hypoesthesia confined to the distribution of the left mental nerve, with no swelling, sinus tract, or tenderness detected. Nonsurgical endodontic retreatment was performed under local anesthesia. Copious 2% sodium hypochlorite irrigation was carried out, followed by calcium hydroxide placement as an interappointment medicament. At the second appointment 2 weeks later, the patient reported complete resolution of the paresthesia. Obturation was completed using warm vertical compaction of gutta‐percha with a bioceramic sealer, followed by coronal restoration. Periapical bone fill and substantial reduction in lesion size were evident at 9 months. Progressive periapical healing was observed at 3 years on periapical and panoramic radiographs. At 5 years, further radiographic resolution was evident, although a small residual radiolucent area remained. At the six‐year follow‐up, CBCT evaluation in the axial, sagittal, and coronal planes demonstrated substantial three‐dimensional bone regeneration and marked reduction of the periapical lesion. The tooth remained functional throughout the follow‐up period, with no recurrence of symptoms.

## 1. Introduction

The inferior alveolar nerve (IAN) and its terminal branch, the mental nerve, are responsible for sensory innervation of the lower lip, chin, and buccal mucosa of the mandibular anterior region [1]. Any pathological process that encroaches upon or compresses these neural structures may result in altered sensation, most commonly manifesting as paresthesia, hypoesthesia, or frank numbness of the lower lip [2].

Although malignant neoplasms and metastatic lesions are classically implicated in the differential diagnosis of lower lip numbness, odontogenic inflammatory conditions represent an underappreciated yet clinically significant etiology [3]. Apical periodontitis is a periradicular inflammatory response triggered by microbial invasion of the root canal system that, when left untreated or inadequately managed, can progress into a chronic destructive lesion of considerable size [4]. As these periapical (PA) lesions expand within the cancellous bone of the mandible, they may eventually approach or directly involve the mandibular canal, exerting mechanical pressure on the neurovascular bundle housed within [5].

The precise mechanism through which large PA lesions induce neural dysfunction is multifactorial. Proposed explanations include direct mechanical compression by the expanding lesion, local ischemia secondary to inflammatory edema, accumulation of purulent exudate within the mandibular bone, and the neurotoxic activity of bacterial byproducts and inflammatory mediators that may compromise neural conduction [6, 7]. Notably, the degree of neurological compromise does not always correlate linearly with lesion dimensions, and some patients remain asymptomatic despite radiographically extensive involvement, whereas others report significant sensory disturbance with comparatively moderate lesion size [8].

Recognizing an odontogenic etiology for lower lip paresthesia carries important clinical implications, as prompt endodontic intervention or surgical management may lead to partial or complete neurological recovery [9]. This report presents a case of lower lip numbness attributable to a large apical periodontitis lesion, with a discussion of its diagnosis, management, and neural recovery outcome.

## 2. Case Presentation

### 2.1. Patient Information

A 30‐year‐old male patient was referred to the endodontic clinic presenting with a chief complaint of numbness and altered sensation involving the lower lip, chin, and buccal mucosa on the left side.

### 2.2. Medical and Dental History

The patient′s general medical history was unremarkable. No history of malignancy, systemic neuropathy, metabolic disorders, or long‐term medication use known to affect peripheral nerve function was reported. From a dental perspective, the patient disclosed a history of previous root canal treatment performed years earlier on tooth 36, though no recent dental intervention had been carried out prior to the onset of symptoms. The patient indicated that the symptoms had gradually developed over a duration of 1 week with no preceding history of trauma, surgical procedure, or dental extraction in the affected region.

### 2.3. Clinical Examination

#### 2.3.1. Extraoral Examination

Extraoral assessment revealed no visible facial asymmetry, soft tissue swelling, or skin changes overlying the affected region. Regional lymph nodes were nonpalpable and nontender. The temporomandibular joint exhibited a normal range of motion.

#### 2.3.2. Intraoral Examination

Intraoral inspection of tooth 36 revealed a defective composite restoration. The surrounding soft tissues appeared healthy. A sinus tract was not identified in the buccal vestibule adjacent to the tooth apex. Periodontal probing depths were within acceptable limits across all measured sites, and no pathological tooth mobility was detected.

#### 2.3.3. Pulp and PA Testing

Because tooth 36 had previously undergone root canal treatment, routine thermal and electrical pulp sensibility testing was not applicable for this tooth. To establish a baseline and rule out adjacent dental pathology, pulp sensibility testing was carried out using refrigerant spray (Endo‐Ice) and an electric pulp tester on the adjacent tooth 35, which responded normally. Vertical and horizontal percussion testing on tooth 36 produced no response. Apical palpation over the buccal vestibule of tooth 36 was unremarkable.

#### 2.3.4. Radiographic Evaluation

A PA radiograph was obtained to assess the status of the periradicular tissues and the previous endodontic therapy of tooth 36. The radiographic evaluation revealed an inadequate previous root canal treatment characterized by poor obturation of the root canals. Furthermore, a complete lack of coronal seal was noted beneath the defective restoration (Figure 1). A large, well‐demarcated PA radiolucency measuring approximately 3 cm in its greatest diameter was observed surrounding the root apices of tooth 36 (Figure 2).

**Figure 1:**
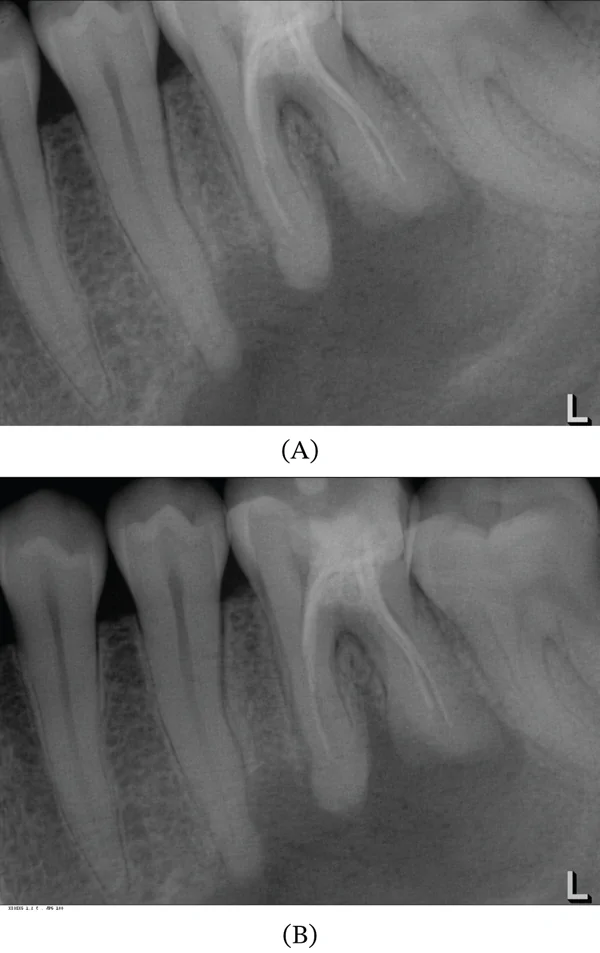
Preoperative periapical radiographs. (A) Initial radiograph showing the previously root‐filled mandibular left first molar and large periapical radiolucency. (B) Radiograph from a different angulation demonstrating the extent of the lesion.

**Figure 2:**
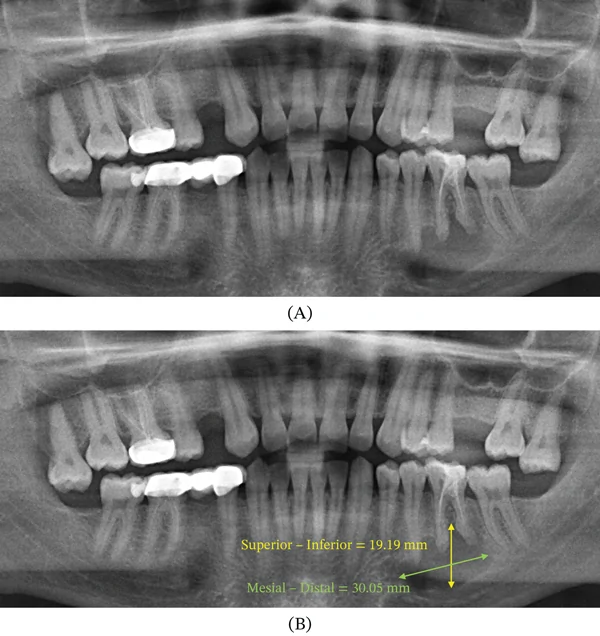
(A) Preoperative panoramic radiograph. (B) Annotated panoramic radiograph showing the dimensions of the lesion.

Based on the clinical and radiographic findings above, the pulpal and PA diagnoses were established as follows:

• Pulpal diagnosis: previously treated
• PA diagnosis: asymptomatic apical periodontitis

#### 2.3.5. Neurosensory Evaluation

Beyond routine clinical examination, a structured neurosensory assessment was conducted to characterize the degree of neural involvement. The patient subjectively reported altered sensation over the lower lip, chin, and buccal mucosa on the left side, with no reported interference with daily functions such as eating or speech. Objective testing was performed following the standardized protocol described by Robinson et al. [10]. The neurosensory findings at baseline and during follow‐up, including comparison with the contralateral unaffected side, are summarized in Table 1. The assessment included the following:

1. Mapping the injury area: Sensory stimuli were systematically applied across the left lower lip, chin, and adjacent mucosa to identify and delineate the boundaries of the hypoesthetic zone. The demarcated area corresponded to the cutaneous distribution of the mental nerve and was documented in the patient′s clinical records (Figure 3).
2. Function scoring: Using a numerical rating scale from 0 (complete absence of sensation) to 10 (fully normal perception), the patient self‐assessed and compared sensation on the affected side against the contralateral unaffected side, scoring 3.
3. Light touch test: A folded paper tip was gently stroked across the affected region and the maneuver was repeated several times. The patient reported diminished tactile perception throughout the area.
4. Sharp/blunt discrimination test: Alternating sharp and blunt stimuli were applied to the affected area using opposite ends of a dental explorer. The patient was partially able to differentiate between the two stimulus types.
5. Two‐point discrimination test: The capacity to distinguish between simultaneous dual‐point contact and single‐point contact was evaluated using open‐beak and closed‐beak tweezer tips. The patient had difficulty identifying the two pressure points as separate stimuli.

**Table 1:** Neurosensory evaluation of the affected and contralateral sides at baseline and during follow‐up.

Neurosensory parameter	Baseline–affected side (left)	Baseline–contralateral side (right)	2 weeks–affected side (left)	9 months–affected side (left)
Subjective sensation	Altered sensation/hypoesthesia	Normal	Normal; paresthesia resolved	Normal; no recurrence
Function score (0–10)	3	10	Normal sensation reported	Normal sensation reported
Light touch	Diminished perception	Normal	Clinically normal	Clinically normal
Sharp/blunt discrimination	Partially impaired	Normal	Clinically normal	Clinically normal
Two‐point discrimination	Difficulty distinguishing two points	Normal	Clinically normal	Clinically normal

*Note:* Neurosensory findings remained within normal limits throughout the 6‐year follow‐up period, with no evidence of sensory deficits or neurological complications during subsequent follow‐up visits.

**Figure 3:**
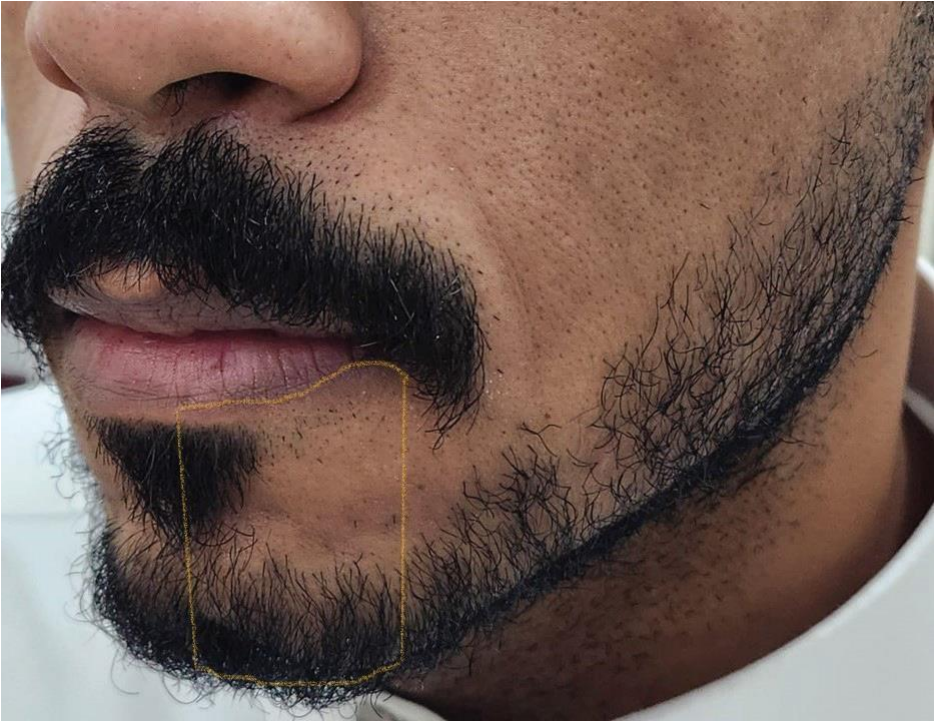
Marked picture showing the numb area.

### 2.4. Treatment Procedure

Prior to initiating any intervention, a thorough discussion was conducted with the patient regarding all available treatment options. These included nonsurgical endodontic retreatment alone, nonsurgical retreatment followed by PA surgery if deemed necessary, or extraction of the tooth with subsequent implant placement. The risks, benefits, and prognosis associated with each option were clearly explained. Given the patient′s preference for a conservative approach and his motivation to preserve the natural tooth, a decision was made to proceed with nonsurgical endodontic retreatment as the initial line of management.

#### 2.4.1. First Visit

Following the acquisition of written informed consent, IAN block anesthesia was administered using mepivacaine 2% with epinephrine 1:100,000 (Scandonest 2% Special, Septodont, Saint‐Maur‐des‐Fossés, France). Rubber dam isolation was placed and the defective composite restoration was removed. Access to the pulp chamber was refined and all canal orifices were identified. The entire procedure was performed by the primary author under 6× magnification using Zumax surgical loupes (Zumax Medical Co. Ltd., Suzhou, China).

Removal of the existing gutta‐percha was carried out using the RACE EVO rotary retreatment system (FKG Dentaire, La Chaux‐de‐Fonds, Switzerland) in a crown‐down manner until the canals were free of the previous obturation material. The mandibular first molar presented with four canals. The mesial root exhibited Vertucci Type IV anatomy, with separate mesiobuccal and mesiolingual canals, whereas the distal root exhibited Vertucci Type V anatomy. Apical patency was confirmed in all four canals using a size 10 K‐file. Working length was established electronically using the J. Morita Root ZX apex locator (J. Morita Mfg. Corp., Kyoto, Japan) and verified radiographically.

Upon achieving patency, spontaneous discharge of purulent exudate was observed from the canal orifices. A microsuction tip was positioned at the canal orifice to aspirate the exudate until discharge ceased completely. Irrigation with 2% sodium hypochlorite (NaOCl) solution (Cerkamed, Stalowa Wola, Poland) was performed using a 30‐gage side‐vented needle positioned 1–2 mm short of the working length, with 2 mL of NaOCl used per canal during each irrigation cycle. The canals were subsequently prepared to a master apical size of ISO 35 with a 4% taper. Following completion of instrumentation and irrigation, the canals were dried with paper points, and calcium hydroxide [Ca(OH)_2_] paste (Ultracal XS, Ultradent Products Inc., South Jordan, Utah, United States) was introduced into all canals as an interappointment intracanal medicament using a lentulo spiral. The access cavity was sealed with a temporary restorative material (Figure 4).

**Figure 4:**
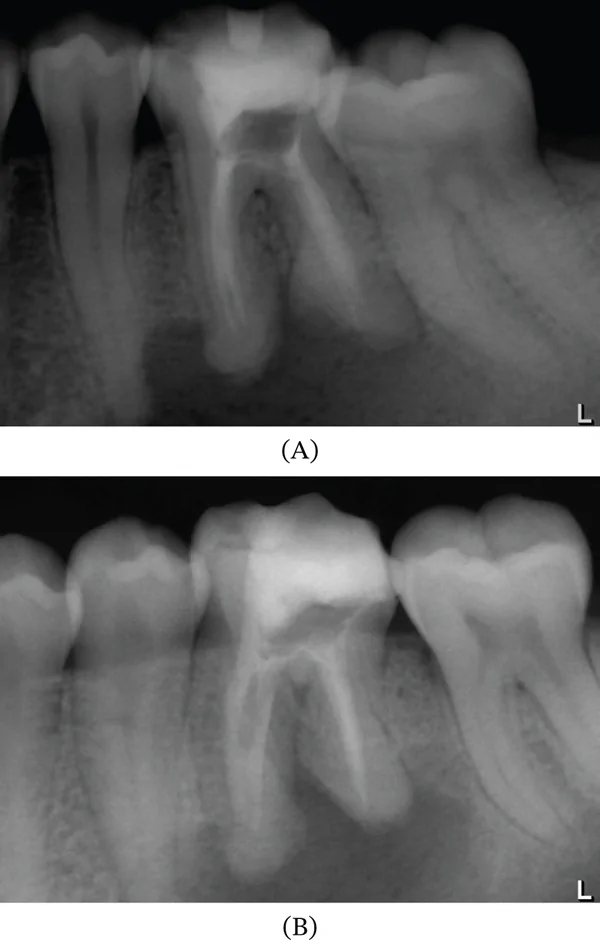
Periapical radiographs after Ca(OH)_2_ placement. (A) Radiograph demonstrating intracanal Ca(OH)₂ placement. (B) Radiograph obtained from a different angulation.

#### 2.4.2. Second Visit—2 Weeks Later

At the follow‐up appointment 2 weeks later, the patient reported complete disappearance of the lower lip paresthesia. Following administration of IAN block anesthesia and placement of rubber dam isolation, the temporary restoration was removed. The Ca(OH)₂ dressing was eliminated from all canals using copious irrigation with 2% NaOCl combined with ultrasonic activation using an endodontic ultrasonic device (VDW Ultra, VDW GmbH, Munich, Germany). Ultrasonic activation was performed in Endo mode for 30 s at the end of the irrigation session. A final irrigation sequence was then performed using 2% NaOCl, followed by 17% ethylenediaminetetraacetic acid (EDTA) solution (Endo‐Solution, Cerkamed, Stalowa Wola, Poland) for 1 min and a final flush with 2% NaOCl. The canals were subsequently dried using sterile absorbent paper points.

Obturation was performed using warm vertical compaction of gutta‐percha in conjunction with a bioceramic (BC) sealer (NeoSealer Flo, Avalon Biomed, Houston, Texas, United States). The master gutta‐percha cones corresponded to the final preparation size 35, 4% taper (Figure 5). The access cavity was restored with glass ionomer cement (Fuji IX, GC Corporation, Tokyo, Japan), and the patient was referred to a specialist colleague in the Advanced Education in General Dentistry (AEGD) program for fabrication of a full‐coverage coronal restoration (Figure 6).

**Figure 5:**
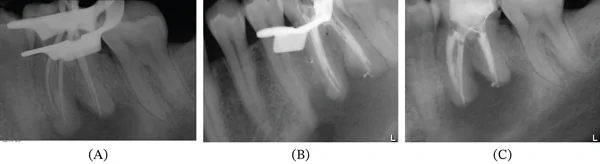
(A) Master apical cone. (B) During obturation. (C) After obturation and restoration by glass ionomer cement.

**Figure 6:**
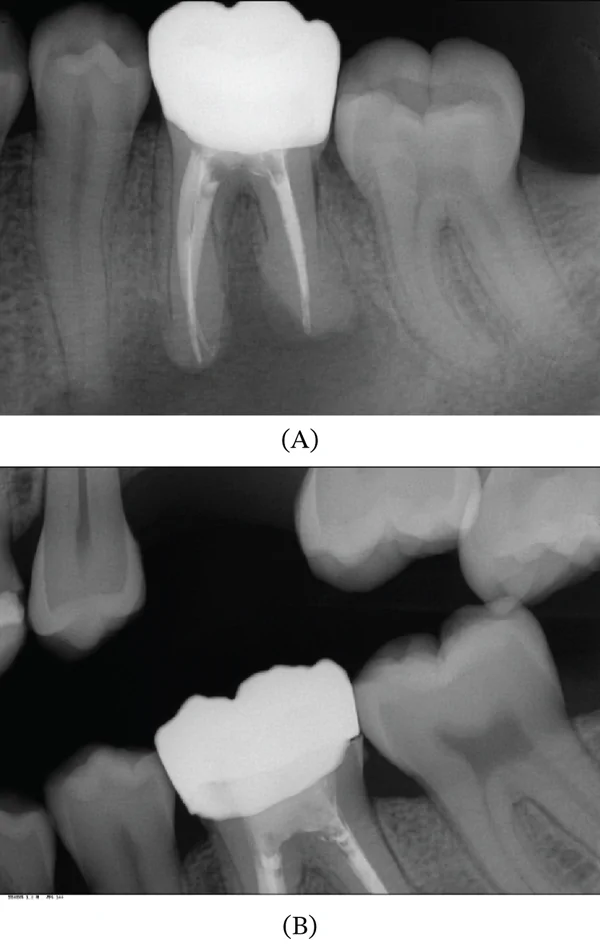
Radiographs after crown placement. (A) Periapical. (B) Bitewing.

### 2.5. Follow Up

The patient was followed up clinically and radiographically over a period of 6 years. Throughout this period, the tooth remained fully functional with no recurrence of pain, swelling, or paresthesia at any follow‐up interval.

A PA radiograph taken at 9 months posttreatment revealed early signs of bone fill at the periphery of the lesion with a notable reduction in the size of the PA radiolucency. At the 3‐year follow‐up, both a PA and panoramic radiograph were obtained, demonstrating continued and significant reduction of the lesion with progressive restoration of bone density in the affected region. Substantial radiographic healing was evident on the PA radiograph taken at 5 years (Figure 7).

**Figure 7:**
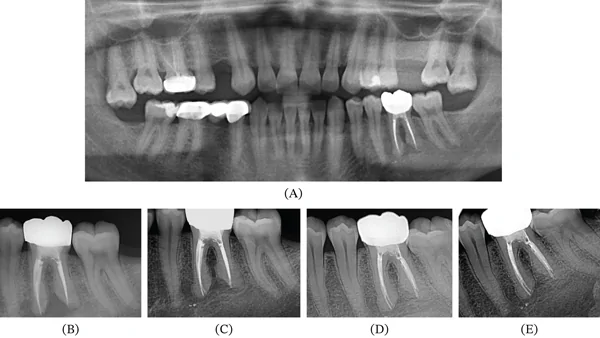
Follow‐up radiographs at different intervals. (A) Panoramic radiograph, 3 years follow‐up. (B) Periapical radiograph, 9 months follow‐up. (C) Periapical radiograph, 3 years follow‐up. (D) Periapical radiograph, 5 years follow‐up. (E) Periapical radiograph, 6 years follow‐up.

At the six‐year follow‐up, a PA radiograph combined with cone beam computed tomography (CBCT) imaging evaluated in three orthogonal planes—axial, sagittal, and coronal—confirmed substantial three‐dimensional resolution of the PA lesion. Cortical plate integrity and trabecular bone architecture were fully restored with no evidence of residual pathology or canal reinfection. The patient remained entirely asymptomatic at this final assessment (Figure 8).

**Figure 8:**
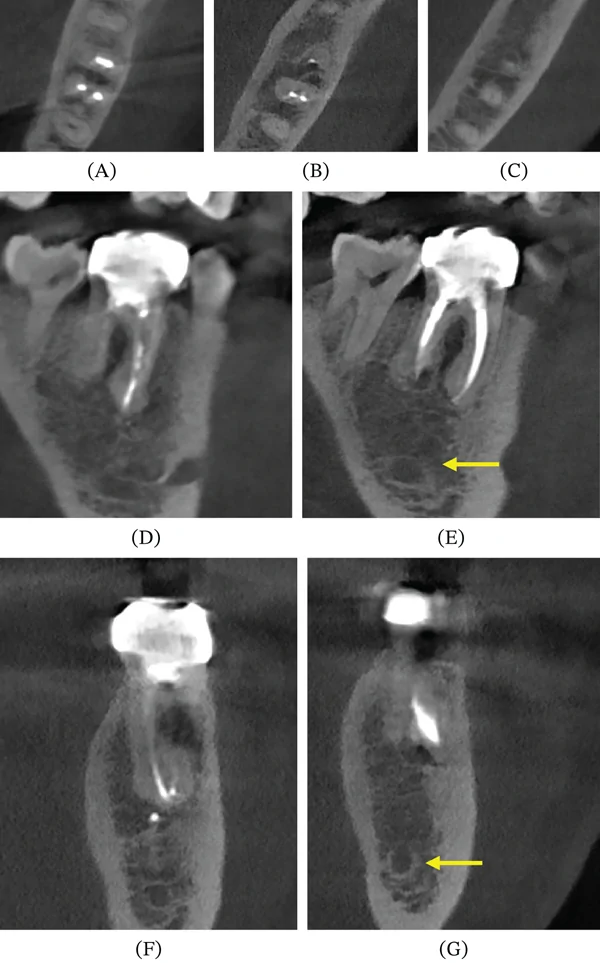
Cone beam computed tomography (CBCT) evaluation at six‐year follow‐up. (A–C) Axial views at sequential levels showing the periapical region circumferentially around the root. (D,E) Sagittal views displaying the three‐dimensional status of the periapical and interradicular bone. (F) Coronal views of the mesial root and (G) distal root showing the periapical bone status at each root individually. Yellow arrows indicate the inferior alveolar canal.

A summary timeline of the diagnostic and treatment course, from initial presentation through the 6‐year follow‐up, is presented in Table 2 to clarify the sequence and duration of interventions.

**Table 2:** Timeline of diagnosis, treatment, and follow‐up.

Time point	Event
Day 0	Patient presents with 1‐week history of left lower lip/chin numbness; clinical exam reveals defective restoration on tooth 36 with pre‐existing root canal treatment; hypoesthesia confirmed in mental nerve distribution
Day 0	Nonsurgical endodontic retreatment initiated under local anesthesia; 2% sodium hypochlorite irrigation; calcium hydroxide placed as interappointment medicament
Week 2	Second appointment; complete resolution of paresthesia reported; obturation completed with warm vertical compaction and bioceramic sealer; coronal restoration placed
9 months	Follow‐up: periapical bone fill and substantial reduction in lesion size
3 years	Follow‐up: progressive periapical healing on periapical and panoramic radiographs
5 years	Follow‐up: further radiographic resolution; small residual radiolucent area remained
6 years	Follow‐up: CBCT shows substantial 3D bone regeneration and marked reduction of periapical lesion; tooth functional, no recurrence of symptoms

## 3. Discussion

The present case illustrates an important yet underrecognized clinical scenario in which large‐scale PA pathology of endodontic origin resulted in sensory disturbance of the mental nerve distribution. Understanding the nature of the nerve injury, its underlying mechanisms, and the rationale for treatment decisions is essential to interpreting the clinical outcome.

From a neurological standpoint, the type of nerve injury most plausibly associated with PA‐induced paresthesia is neuropraxia—the mildest category within Seddon′s classical three‐tier classification of peripheral nerve injuries, which also includes axonotmesis and neurotmesis. In neuropraxia, nerve impulse conduction is blocked at the site of injury, with partial or complete loss of motor and sensory function, although all structural components of the nerve—including the endoneurium, perineurium, and epineurium—remain anatomically intact. Importantly, neuropraxic injuries generally carry a favorable prognosis, with spontaneous recovery anticipated once remyelination is complete, typically within 3 months. This classification is particularly relevant in endodontic‐related paresthesia, where neural injury is reversible once the causative pathology is adequately resolved [1].

Paresthesia of the lower lip may arise from a broad spectrum of etiological factors, including systemic conditions such as multiple sclerosis, hematological malignancies, viral neuropathies, and metastatic disease, as well as local dental causes such as IAN block injections, surgical trauma, and overfilled root canal materials [2]. Among local odontogenic etiologies, the mechanism of paresthesia induction due to PA infection is considered multifactorial, encompassing local mechanical pressure from accumulating purulent exudate within the mandibular bone, the neurotoxic activity of inflammatory bacterial byproducts, and in some instances, direct bacterial invasion of neural structures [11]. In the present case, the proximity of the expanding PA lesion to the mandibular canal may have contributed to a compressive and inflammatory insult to the IAN and its mental branch, consistent with previously documented cases of endodontic‐origin mental nerve neuropathy [8, 12].

A limitation of this case is that a pretreatment CBCT was not available to objectively assess the spatial relationship between the lesion and the mandibular canal or to demonstrate direct nerve contact or compression. Moreover, alternative mechanisms, including diffusion of inflammatory mediators or bacterial by‐products to the nerve without direct physical contact, cannot be excluded. Therefore, the association between the PA lesion and mental nerve paresthesia, and the proposed underlying mechanism, should be considered a plausible clinical attribution rather than a definitively demonstrated causal relationship.

Successful elimination of the bacterial load from the root canal system represents the cornerstone of PA healing and, consequently, neural recovery. NaOCl remains the irrigant of choice in root canal therapy owing to its broad‐spectrum antimicrobial activity and capacity for organic tissue dissolution. Studies have demonstrated that concentrations of 2.5% and 5% NaOCl effectively eliminate *Enterococcus faecalis* biofilms across different stages of maturation, whereas lower concentrations show progressively reduced efficacy against older and more resilient biofilms [13].

Managing active purulent discharge through the root canal system presents a distinct clinical challenge. Historically, teeth with persistent exudation were occasionally left open to the oral cavity—a practice now heavily discouraged due to the risk of secondary microbial contamination and biofilm complication. In the present case, a definitive closed‐system management protocol was maintained by utilizing an endodontic microsuction tip to actively evacuate the purulent tract [14]. This technique aligns with the principles of active nonsurgical decompression and vacuum‐assisted aspiration described in the literature. By generating localized negative pressure within the canal lumen, microsuction safely and effectively decompresses the PA lesion. This immediate mechanical evacuation reduces interstitial tissue pressure within the bone, arresting the compressive and neurotoxic effects on the adjacent mental nerve bundle and setting the stage for accelerated neural recovery [15].

Complementing irrigation, Ca(OH)₂ was employed as an interappointment intracanal medicament. Clinical evidence has shown that a minimum 7‐day application of Ca(OH)₂ as an intracanal dressing effectively eliminates bacteria surviving chemomechanical instrumentation, whereas shorter contact times prove insufficient [16]. The use of a multiple‐visit protocol in this case was deliberate, allowing adequate time for microbial suppression and PA decompression prior to obturation [17].

For final obturation, a BC sealer was selected because of its favorable biological and antimicrobial profile. A systematic review evaluating PA healing outcomes following endodontic treatment with different sealer types reported that, although PA lesions demonstrated significant healing regardless of sealer type, BC and bioactive sealers consistently produced superior results [18]. The bioactivity of BC sealers is attributed to their calcium silicate composition, which upon contact with PA tissue fluids promotes the formation of hydroxyapatite—a process that facilitates chemical bonding with dentinal walls and creates a favorable biological environment for tissue regeneration. These properties are particularly advantageous in cases of necrotic pulp or retreatment, where the risk of residual bacterial contamination is higher [19]. Furthermore, a 12‐month observational study evaluating a tricalcium silicate BC sealer reported that PA index scores decreased significantly over time in apical periodontitis cases, with a 97% clinical success rate and all treated teeth remaining asymptomatic and functional throughout the follow‐up period [20]. These cumulative biological properties make BC sealers a scientifically sound choice in cases where PA healing and neural recovery are the primary treatment objectives.

The long‐term radiographic outcome of this case was further substantiated by 6‐year CBCT evaluation. CBCT was performed to provide a three‐dimensional assessment of the healing status, as conventional two‐dimensional radiographs may not accurately reflect the actual extent of residual osseous defects and may give an appearance of complete or near‐complete healing despite persistent areas of bone loss. Although axial, sagittal, and coronal views confirmed substantial three‐dimensional PA healing with restoration of cortical plate integrity and peripheral bone density, a relative reduction in bone density was noted in the interradicular region and around the distal root apex compared with the surrounding bone. Notably, the inferior alveolar canal demonstrated no evidence of encroachment, compression, or pathological changes in its immediate vicinity, with the surrounding bone appearing well healed and the canal boundaries clearly intact. Thus, the CBCT findings provided additional three‐dimensional information that could not be fully appreciated on the conventional two‐dimensional radiographs. This restoration of normal bone architecture in the proximity of the inferior alveolar canal is consistent with the complete resolution of the patient′s paresthesia observed as early as the second clinical visit. The residual hypodensity in certain regions likely reflects incomplete bone maturation rather than treatment failure, as large PA lesions are known to undergo a slow and progressive healing process, with CBCT frequently revealing areas of residual bone hypodensity not detectable on conventional two‐dimensional radiographs [21]. Moreover, different anatomical compartments within a large lesion may heal at varying rates, with some regions demonstrating radiographic resolution earlier than others [22]. The absence of clinical symptoms, combined with the overall pattern of progressive bone density restoration observed across all follow‐up intervals, supports an ongoing healing trajectory rather than pathological recurrence.

The preoperative CBCT scan was unavailable because of technical reasons, which precluded direct three‐dimensional volumetric comparison of the lesion before and after treatment. However, preoperative PA radiographs documented the baseline lesion, and sequential PA radiographs combined with 6‐year CBCT evaluation collectively confirm progressive and substantial PA healing. Additionally, as with all case reports, findings represent a single clinical scenario and cannot be generalized to the broader patient population.

## 4. Conclusion

This case highlights that large PA lesions of endodontic origin can present with mental nerve paresthesia and should be considered in the differential diagnosis of lower lip and chin numbness alongside malignant causes. Timely recognition of the odontogenic source allowed for nonsurgical endodontic retreatment rather than more invasive intervention. The neurosensory disturbance resolved rapidly following disinfection and intracanal medication, whereas the PA lesion showed progressive radiographic healing over an extended follow‐up period, with substantial bone regeneration confirmed on CBCT at 6 years. This case reinforces nonsurgical root canal retreatment as an effective, tooth‐preserving option even for large PA lesions.

## Funding

No funding was received for this manuscript.

## Disclosure

The author was responsible for the clinical management of the patient, data collection and interpretation, preparation and revision of the manuscript, and final approval of the submitted version.

## Ethics Statement

The study received an exemption from ethics approval by the Institutional Review Board of Riyadh Elm University.

## Consent

Written informed consent was obtained from the patient for the publication of this case report, including all clinical findings, radiographic images, and follow‐up data.

## Conflicts of Interest

The author declares no conflicts of interest.

## Supporting information


**Supporting Information** Additional supporting information can be found online in the Supporting Information section. Data S1: CARE checklist of information to include when writing a case report.

## Data Availability

All data relevant to this case report are included in the published article.
